# Job Design for Home Care Work: Perspectives From Employers and Home Care Aides

**DOI:** 10.1093/geroni/igab046.834

**Published:** 2021-12-17

**Authors:** Natasha Bryant, Robyn Stone, Alex Hennessa

**Affiliations:** 1 LeadingAge LTSS Center @UMass Boston, Washington, District of Columbia, United States; 2 LeadingAge, Washington, District of Columbia, United States

## Abstract

Home-based care is a rapidly growing sector becoming more important to individuals, families, providers, and payers. The ways in which agencies create the work environment for home care aides who are essentially in their clients’ homes is not adequately documented and may be changing rapidly with labor market innovations. This qualitative study describes how different home care business models (e.g., non-profit VNAs, for-profit franchises, uber-style matching, worker-owned coops) address job design and the overall work environment for home care aides. Interviews with employers and focus groups with home care aides examine workplace practices, how work is organized and supported when the workforce is virtual and the workplace is a client’s home, and the perceived attributes of a positive workplace environment across business models. This study fills significant knowledge gaps about home care workplace design and the role of agencies in creating a supportive environment.

